# Dual-suture fundoplication for anti-reflux reconstruction after proximal gastrectomy: a single-center retrospective case series

**DOI:** 10.1186/s12893-026-03499-7

**Published:** 2026-01-26

**Authors:** Yu-Xuan Yan, Huai-Ping Cui, Ji-Zhun Zhang, Zhu Wang, Qin-Hui Sun, Li-Tao Tian, Ze-Xin Wang, Chuan-Zheng Yang, Jin-Shen Wang

**Affiliations:** 1 Department of Gastrointestinal Surgery, Shandong Provincial Hospital Affiliated to Shandong First Medical University, 324 Jingwu Weiqi Road, Huaiyin District, Jinan, Shandong 250021 China; 2 Department of Gastrointestinal Surgery, Zibo Central Hospital, Zibo, Shandong 255036 China; 3 Gastrointestinal Surgery, The Third Affiliated Hospital of Shandong First Medical University, Jinan, Shandong 250031 China

**Keywords:** Laparoscopic gastrectomy, Proximal gastrectomy, Esophagogastrostomy, Gastroesophageal reflux, Surgical technique, Simplified reconstruction

## Abstract

**Background:**

Esophagogastrostomy (EG) after proximal gastrectomy (PG) is widely used but often complicated by reflux. Current anti-reflux procedures, such as double-tract and double-flap reconstructions, are effective but technically demanding. We developed a simplified, device-free anti-reflux EG that uses two sutures to approximate the angle of His, the gastric angle, and a neofundus-like contour.

**Methods:**

We retrospectively analyzed 11 consecutive patients with upper-third gastric cancer who underwent laparoscopic PG followed by dual-suture fundoplication between May 2023 and November 2024. Surgical and clinical outcomes included operative time, blood loss, hospital stay, complications (Clavien–Dindo), reflux symptoms, endoscopic findings, and quality of life assessed using the Reflux Disease Questionnaire (RDQ) and the World Health Organization Quality of Life-BREF (WHOQOL-BREF). Continuous variables were summarized as mean ± standard deviation and range. Changes in RDQ and WHOQOL-BREF scores were compared using paired tests (paired t-test or Wilcoxon signed-rank test after normality assessment), with two-sided α = 0.05.

**Results:**

All procedures were successfully completed without intraoperative or postoperative complications (Clavien–Dindo). The mean operative time was 189.9 min and the mean hospital stay was 7.4 days. During a median follow-up of 12 months, no patient required proton pump inhibitors, and no reflux esophagitis of Los Angeles grade B or higher was observed. RDQ scores remained stable, while WHOQOL-BREF scores were higher in the social and environmental domains, suggesting favorable postoperative function and quality of life.

**Conclusion:**

This simplified dual-suture esophagogastrostomy appeared safe and feasible in this cohort and demonstrated reassuring early postoperative outcomes with respect to reflux. Its minimal technical demands and favorable postoperative recovery profile suggest potential suitability for broader clinical application, although larger comparative studies with extended follow-up are needed to clarify long-term outcomes.

**Supplementary Information:**

The online version contains supplementary material available at 10.1186/s12893-026-03499-7.

## Introduction

Over the past five decades, despite a global decline in the incidence of gastric cancer [1], the incidence and mortality rates of esophageal adenocarcinoma and adenocarcinoma of the esophagogastric junction (AEG) remain notably high, particularly in East Asian populations [2–4]. AEG is a malignant tumor occurring at the esophagogastric junction, possessing unique biological characteristics distinct from both esophageal and gastric cancers. Its special anatomical location at the transition zone between squamous and columnar epithelium presents challenges in defining optimal surgical treatment strategies, which remain a subject of ongoing debate [5].

Currently, surgery remains the cornerstone of treatment for AEG [6–8]. Proximal gastrectomy (PG) has emerged as a preferred surgical option for Siewert type II AEG and select upper gastric cancers, owing to its ability to preserve gastric function and improve postoperative nutrition and quality of life [9–11]. However, effective reconstruction of the digestive tract after PG remains a critical challenge, particularly in preventing postoperative gastroesophageal reflux disease (GERD) [12]. Digestive tract reconstruction following PG has thus become a critical area of research. Among various reconstruction methods, esophagogastrostomy (EG) is frequently used because of its technical simplicity, preservation of the physiological food passage, and cost-effectiveness. Nevertheless, conventional EG is often limited by a high incidence of postoperative reflux, which can impair patient outcomes and quality of life.

Recent studies have underscored the importance of anatomical structures such as the angle of His, gastric angle, and fundus in maintaining the anti-reflux barrier [13–15]. Based on these principles, we developed a novel, biomimetic EG reconstruction technique that simultaneously restores these key anatomical landmarks to enhance anti-reflux function. Importantly, this method requires only two key sutures, does not rely on specialized instruments, and can be completed in less time than traditional anti-reflux procedures. Its technical simplicity, reproducibility, and safety make it particularly suitable for implementation in primary and secondary care centers.

## Patients and methods

### Patients

This study was designed as a retrospective single-arm case series to evaluate the safety, feasibility, and early outcomes of a simplified anti-reflux esophagogastrostomy following laparoscopic proximal gastrectomy. Eleven patients (7 males and 4 females) who underwent the modified anastomosis technique at Shandong Provincial Hospital Affiliated to Shandong First Medical University between May 2023 and November 2024 were included (Table 1). The cohort had a median age of 64 years (52–75) and a mean BMI of 24.7 kg/m² (20.8–29.1). All tumors were located in the upper third of the stomach and were preoperatively diagnosed by endoscopy, upper gastrointestinal contrast imaging, and computed tomography. Tumor staging, based on the 8th edition of the Union for International Cancer Control (UICC)/American Joint Committee on Cancer (AJCC) TNM classification, included 5 cases of stage IA and 2 cases each of stages IB, IIA, and IIB. No patient had evidence of distant metastasis.

**Table 1:** Patient demographics and surgical outcomes

Clinicopathological characteristics	*N* = 11
Age, mean(years)	62.8 ± 7.7(52–75)
Sex(M/F)	7/4
Body Mass Index(BMI), mean༈（kg/m^2^༉）	24.7 ± 2.7(20.8–29.1)
TNM stage distribution	
IA	5
IB	2
IIA	2
IIB	2
Operation time, mean(min)	189.9 ± 40.9(125–257)
Reconstruction time, mean(min)	54.9 ± 8.5(40–68)
Blood loss, mean (ml)	49.4 ± 12.4(35–75)
Postoperative complications (Clavien–Dindo classification)	0
RDQ score, median(IQR)	
Preoperative	2(1–2)
Postoperative 6-month	2(1–3)
Median change (Bootstrap 95% CI)	0(0–1)
*P*-value (Wilcoxon signed-rank test)	0.102
Endoscopic findings	
Reflux esophagitis	0
Anastomotic stenosis	0
Mortality	0
Postoperative hospital stay, mean (day)	7.4 ± 0.9(6–8)

Inclusion criteria were as follows: histologically confirmed upper-third gastric cancer or Siewert type III adenocarcinoma of the esophagogastric junction; early-stage or T2–T3 non-diffuse type tumors with a maximum diameter < 5 cm; age ≤ 75 years; and adequate organ function to tolerate laparoscopic surgery. Patients were excluded if they had diffuse-type or lower-third tumors, a tumor diameter ≥ 5 cm, T4a or more advanced disease, suspected metastases to lower perigastric lymph nodes (stations No. 4 d, 5, or 6), contraindications to general anesthesia, or incomplete clinical records. All patients provided written informed consent for the surgical procedure and for the use of anonymized clinical data in research. The study protocol was approved by the institutional ethics committee and conducted in accordance with the principles of the Declaration of Helsinki.

### Surgical procedure

#### Proximal gastrectomy

After satisfactory induction of general anesthesia, the patient is placed in the supine position with the legs apart, and the surgical field is routinely sterilized and draped. A urinary catheter is inserted, and the patient is positioned in a reverse Trendelenburg position with a slight left tilt to facilitate exposure of the upper abdomen.

**Fig. 1:**
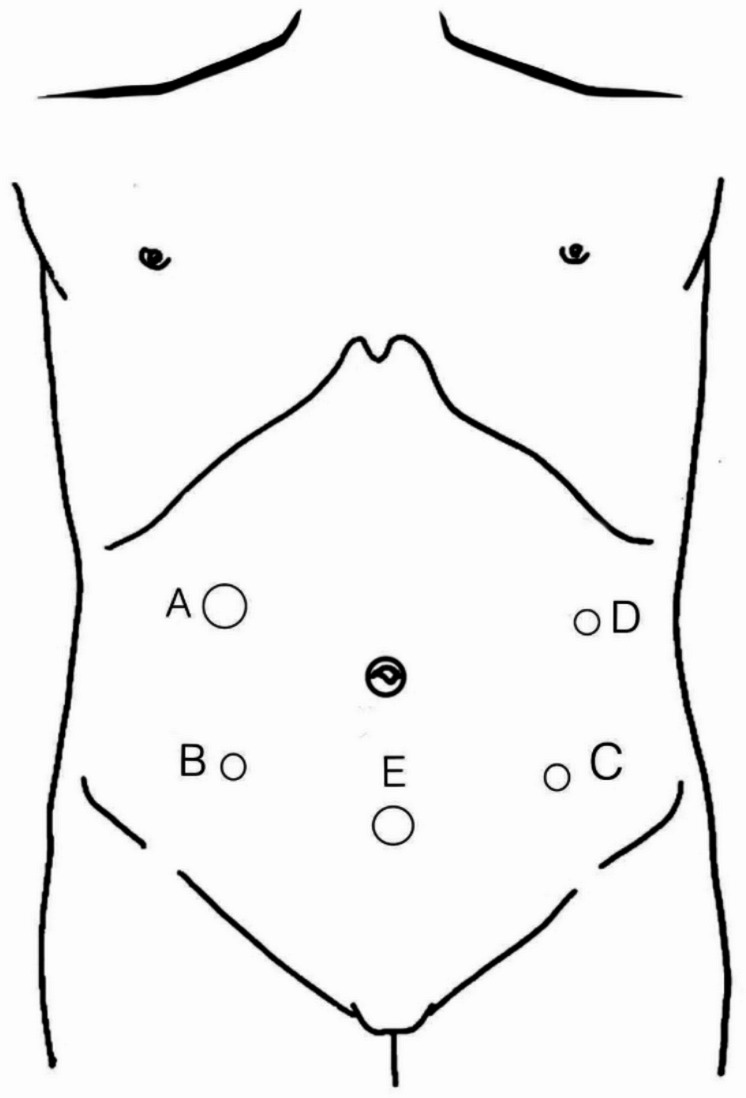
Trocar sites for laparoscopic proximal gastrectomy **A** 12 mm trocar, **B**,**C**, **D** 5 mm trocar site, **E** laparoscope port

A 10 mm laparoscopic trocar is introduced through a vertical incision approximately 2 cm below the umbilicus to establish a pneumoperitoneum (12–14 mmHg). The laparoscope is inserted, and four additional trocars are placed under direct vision: one 12-mm trocar in the right lower quadrant (surgeon’s main port), one 5-mm trocar in the left lower quadrant (assistant’s port), and two 5-mm trocars along the left and right anterior axillary lines for the surgeon and assistant (Fig. 1). Lymph node dissection is performed in accordance with the Japanese gastric cancer treatment guidelines (JGCTG, 2010, version 3; 2014, version 4) [16]. The greater omentum is elevated, and the gastrocolic ligament is divided to access lymph nodes along the short gastric vessels (station #4sa) and near the splenic hilum (station #4sb). Dissection extends along the right gastroepiploic artery to clear station #6 lymph nodes. The lesser omentum is opened to expose and remove lymph nodes along the lesser curvature (stations #3 and #5) and at the esophagogastric junction (stations #1 and #2).

The left gastric artery is exposed and ligated at its origin, allowing removal of lymph nodes from stations #7, #9, and #8a. The anterior leaflet of the transverse mesocolon is lifted to dissect nodes along the splenic artery (stations #11p and #11d). Finally, the esophagus is mobilized approximately 2–3 cm above the esophagogastric junction to ensure complete clearance of periesophageal lymph nodes.

A small upper midline incision (5–8 cm) is made to exteriorize the specimen. The esophagus is transected 2 cm above the tumor after applying a purse-string suture, and the anvil of a circular stapler is inserted into the esophageal stump. The stomach is transected 5 cm distal to the tumor margin using a linear stapler. The specimen is removed, and the gastric remnant is reinforced with interrupted sutures.

### Two-suture biomimetic esophagogastrostomy

**Fig. 2:**
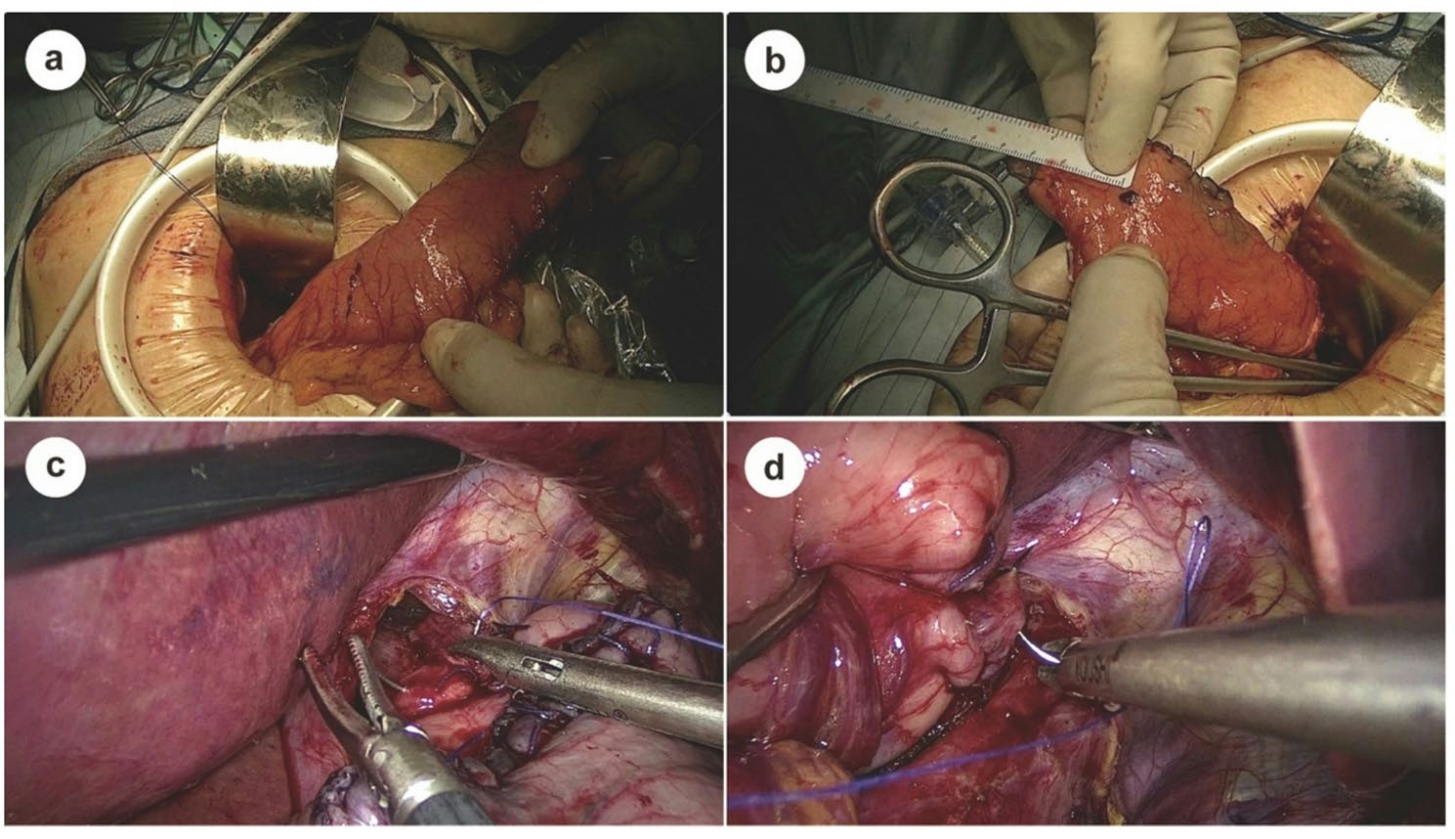
Key intraoperative steps of the two-suture biomimetic esophagogastrostomy. **a** Anterior gastric wall incision made 4–5 cm from the pylorus for stapler insertion. **b** Posterior gastric wall punctured approximately 3 cm below the gastric stump apex for stapler insertion. **c**, **d** Seromuscular sutures on both sides of the esophagus to the remnant stomach to reconstruct the angle of His and artificial fundus

**Fig. 3:**
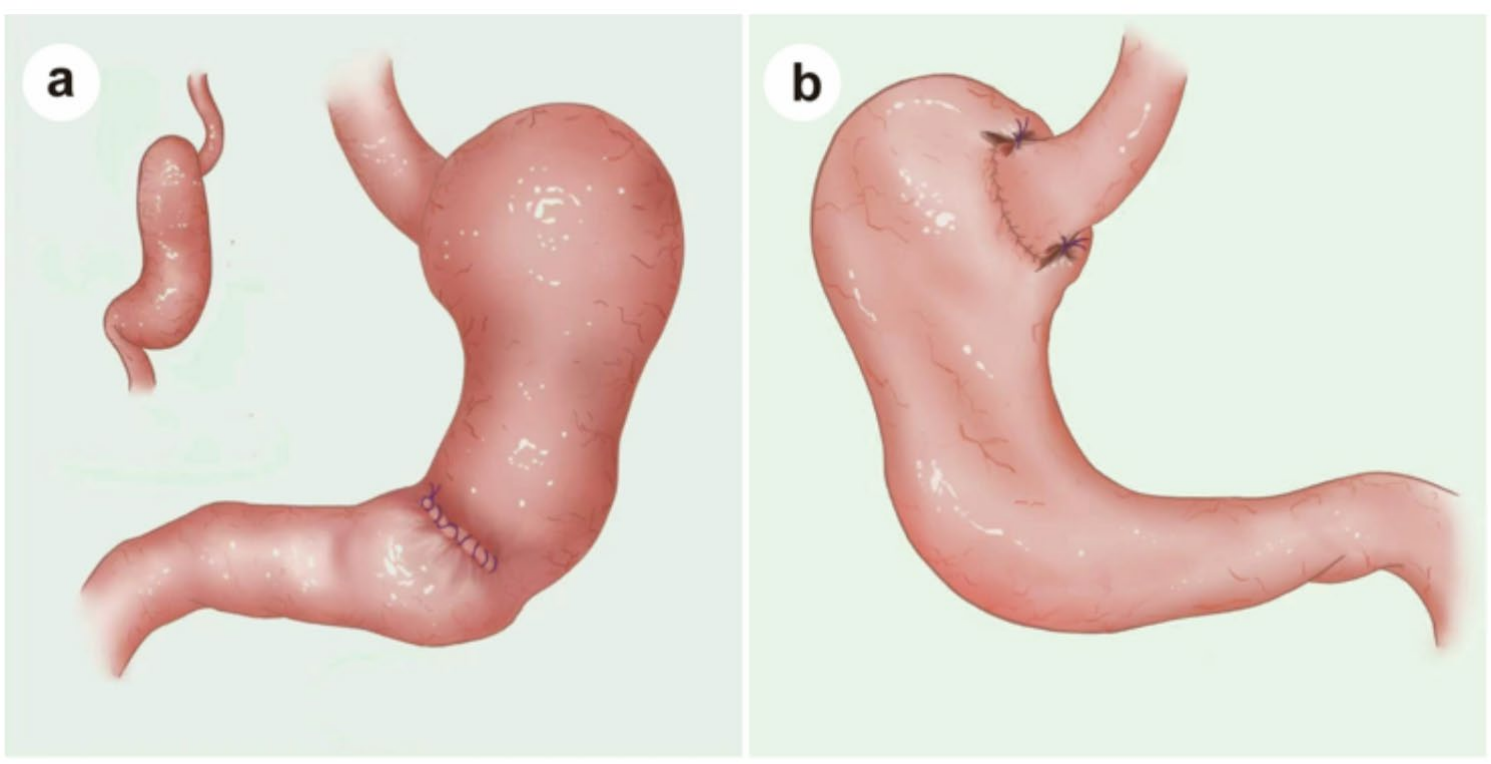
Intraoperative schematic of simplified anti-reflux reconstruction. **a** Anterior and lateral views: A side-to-end esophagogastrostomy is constructed with full-thickness reinforcement sutures around the anastomosis. **b** Posterior view: Two key seromuscular sutures are placed between the left and right sides of the esophagus and the remnant stomach, reconstructing the angle of His and forming a functional neofundus, as later confirmed by endoscopy

**Fig. 4:**
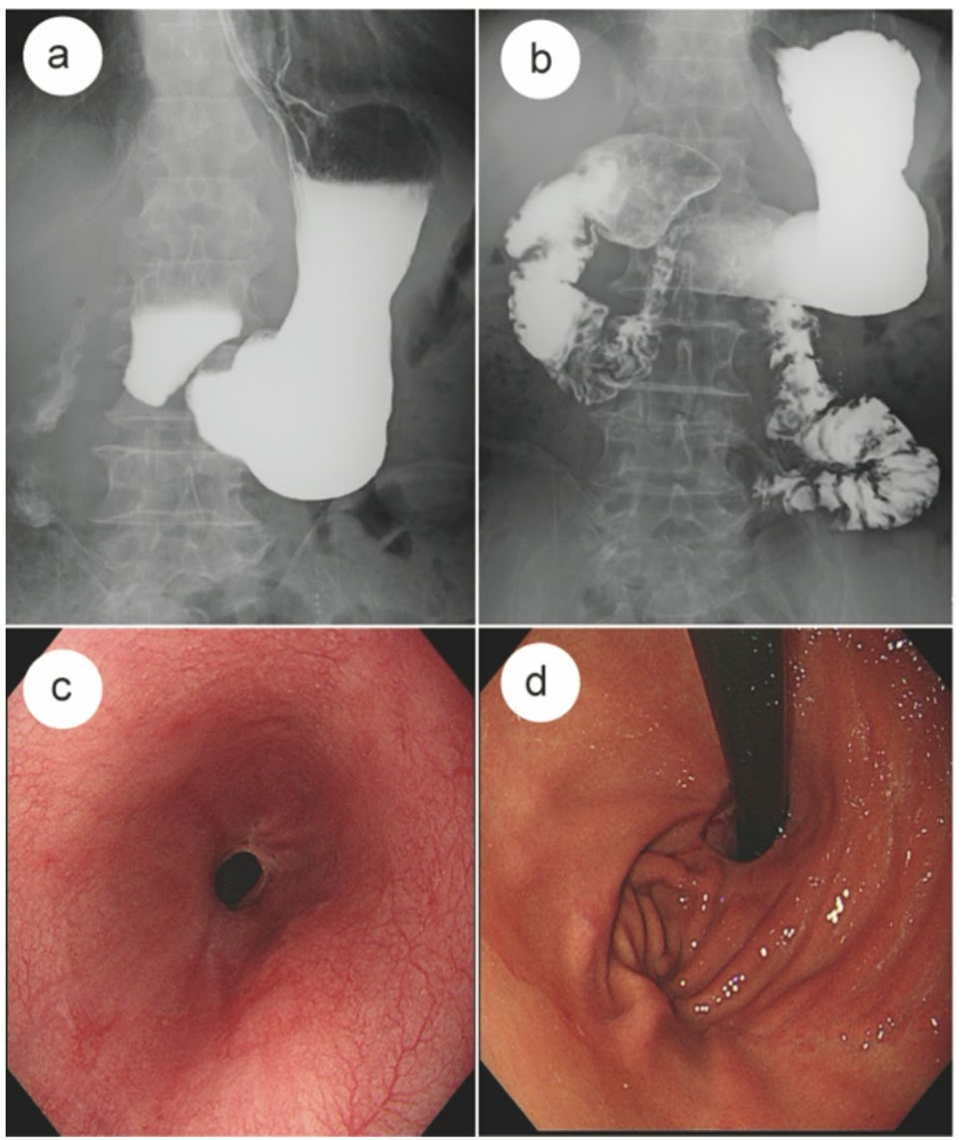
**a**, **b** Upper gastrointestinal iodinated contrast examination at 6 months postoperatively. **a** A clear His angle and gastric angle are visible, and the proximal gastric contour shows partial restoration of an angular configuration. **b** The anastomotic site appears smooth and well-outlined, with no contrast reflux observed. The contrast medium passes readily into the distal small intestine. **c**,** d** Endoscopic examination at 6 months postoperatively. **c** The esophagogastric anastomosis shows intact mucosal continuity, a smooth lumen, and no erosive changes suggestive of reflux. **d** A small fold-like structure resembling a neofundus is observed at the reconstructed gastroesophageal junction

After preparing the esophageal stump and securing a purse-string suture, the anvil of a circular stapler is inserted and tightly fixed into the stump. A transverse incision is made on the anterior wall of the gastric remnant, approximately 4–5 cm proximal to the pylorus, to serve as the entry point for the stapler (Fig. 2a). The posterior wall of the gastric remnant is perforated about 3 cm below the gastric stump apex (Fig. 2b), allowing the stapler head to exit and connect with the esophageal anvil. A side-to-end esophagogastrostomy is subsequently performed, with the anastomotic diameter controlled within 2.5–3.0 cm to restore gastrointestinal continuity, followed by full-thickness suturing to reinforce the esophagogastric anastomosis circumferentially. To optimize anti-reflux functionality, the anterior wall incision is carefully closed with interrupted seromuscular sutures, and the incision ends are reinforced with imbricating sutures to shorten the anterior wall relative to the posterior wall, forming a physiological gastric angle, as confirmed by postoperative imaging (Figs. 3, 4a and b).

Following anastomosis, two additional key sutures are placed to establish an effective anti-reflux barrier. The first suture anchors the seromuscular layer of the gastric base, approximately 1 cm below the anastomosis, to the right muscular layer of the esophagus, contributing to the formation of an anatomical configuration associated with resistance to reflux around the anastomosis, while also helping to stabilize the anastomotic configuration and limit excessive rotation of the remnant stomach (Fig. 2c). The second suture fixes the stump apex, approximately 1 cm above the anastomosis, to the left muscular layer of the esophagus, thereby reconstructing the angle of His and forming a functional neofundus, as confirmed by postoperative endoscopy (Figs. 2d and 4d). This simplified two-suture technique mimics key anatomical features of a physiological anti-reflux valve by forming an acute angle (~ 80°) and establishing a localized anatomical constraint, while providing dynamic support through fundal compression during gastric filling. The entire process adds less than 5 min to the operative time, requires no special instruments, and imposes no additional tension on the anastomosis.

### Outcome measures

Clinical assessments were performed at baseline and again at 6 months postoperatively according to protocol. Baseline assessments were obtained during the index admission within 1–3 days before surgery. Surgical outcomes included operative time, reconstruction time, intraoperative blood loss, postoperative hospital stay, and complications graded using the Clavien–Dindo classification, with 30-day readmission and reintervention events also recorded. Reflux was assessed endoscopically using the Los Angeles classification and symptomatically using the validated Reflux Disease Questionnaire (RDQ; score range 0–40; GERD threshold ≥ 12). Health-related quality of life was evaluated using the validated WHOQOL-BREF across its physical, psychological, social, and environmental domains. When available, upper gastrointestinal contrast studies were used to document the neo-His angle, gastric angle, neofundus contour, and reflux column height.

### Statistical analysis

All analyses were conducted using SPSS version 26.0 (IBM Corp., Armonk, NY, USA). The distribution of continuous variables was evaluated with the Shapiro–Wilk test. Variables with normal distribution are reported as mean ± standard deviation (SD) and were compared using paired t-tests. Non-normally distributed variables, including RDQ scores, are presented as median with interquartile range (IQR) and were analyzed using the Wilcoxon signed-rank test. Exact two-sided P values and 95% confidence intervals (CIs) were provided when applicable. Statistical significance was defined as *P* < 0.05.

## Results

The surgical outcomes and postoperative recovery of the 11 patients are summarized in Table 1. All 11 patients completed the scheduled baseline and 6-month postoperative assessments without loss to follow-up. All patients underwent laparoscopic proximal gastrectomy with D1 + or D2 lymph node dissection according to JGCTG ver.3 and ver.4 (2010; 2014). The mean operative time was 189.9 min (range, 125–257), including 54.9 min (range, 40–68) for gastrointestinal reconstruction. The estimated mean intraoperative blood loss was 49.4 ml (range, 35–75), and the mean postoperative hospital stay was 7.4 days (range, 6–8). All patients recovered uneventfully without postoperative complications (Clavien–Dindo) and there were no 30-day readmissions or reinterventions. During a median follow-up of 12 months (range, 7–25), none reported severe reflux symptoms such as heartburn or chest pain, and no proton pump inhibitors were required. Postoperative endoscopy in 11 patients revealed no cases of reflux esophagitis classified as Los Angeles grade B or higher (Fig. 4c). Gastroesophageal reflux was further assessed with the RDQ at baseline and at 6 months postoperatively. Shapiro–Wilk testing indicated that the differences between pre- and postoperative scores were not normally distributed (*P* = 0.006); Therefore, the Wilcoxon signed-rank test was used. The median RDQ score was 2.0 (1.0–2.0) preoperatively and 2.0 (1.0–3.0) at 6 months postoperatively, with no statistically significant difference (*Z* = −1.633,*P* = 0.102), indicating that no notable increase in reflux symptoms occurred following the digestive tract reconstruction.

In addition, patient-reported quality of life was evaluated using the WHOQOL-BREF questionnaire at 6 months postoperatively. Compared with baseline, scores in social relationships (11.51 ± 2.37 vs. 10.12 ± 2.21, *P* = 0.039) and environmental domains (10.78 ± 0.82 vs. 9.95 ± 0.72, *P* = 0.049) were higher at follow-up. Although physical and psychological health scores also increased, these changes were not statistically significant. Taken together, these results suggest a possible trend toward improved postoperative quality of life after the modified EG reconstruction, although the findings should be interpreted with caution given the limited sample size (Table 2, Supplementary Fig. 1).

**Table 2:** Preoperative and postoperative health-related quality of life assessed by WHOQOL-BREF

Domain	Pre-op	Post-op(6 m)	Difference (mean, 95% CI)	t	*P*-value
Physical health	10.03 ± 1.18	10.86 ± 1.73	0.83 (−0.07 to 1.73)	2.05	0.067
Psychological health	11.45 ± 1.29	11.82 ± 1.43	0.36 (−0.56 to 1.29)	0.88	0.401
Social relationships	10.12 ± 2.21	11.51 ± 2.37	1.39 (0.09 to 2.70)	2.38	0.039
Environment	9.95 ± 0.72	10.78 ± 0.82	0.82 (0.01 to 1.63)	2.24	0.049

* *WHOQOL-BREF* World Health Organization Quality of Life-BREF

Values are presented as mean ± standard deviation. Paired t-test was used for comparison. Statistical significance was defined as *P* < 0.05

## Discussion

As public awareness of health issues increases and diagnostic technologies improve, the detection of early gastric cancer and early AEG is expected to rise. At present, surgery remains the primary treatment for upper gastric cancer [7, 17, 18], and PG has been increasingly adopted in East Asia because it offers better postoperative nutritional outcomes and fewer symptoms such as diarrhea compared with TG (*P*<0.05) [19].

Conventional esophagogastrostomy (EG) is widely used after PG because it requires only a single anastomosis and facilitates postoperative endoscopic surveillance [20]. Its main limitation lies in the reconstruction of the anti-reflux barrier: the loss of the lower esophageal sphincter and the removal of structures such as the fundus, angle of His, and gastric angle weaken the natural flap-valve mechanism, making reflux more likely when intragastric pressure rises [21]. To address this limitation, several anti-reflux reconstructions have been developed, each with distinct strengths and drawbacks. The double-flap technique (DFT) provides reliable reflux control through a submucosal valve mechanism, but its meticulous flap creation and multilayer suturing increase operative complexity and limit reproducibility [15]. Double-tract reconstruction (DTR) avoids excessive reflux by diverting part of the food stream distally, yet the multiple anastomoses required inevitably prolong operative time and involve additional small-bowel handling [22]. Jejunal interposition (JPI) offers a buffer segment that reduces refluxate exposure, but its multi-step construction and variable postoperative motility reduce its practicality in routine settings [20].

**Fig. 5:**
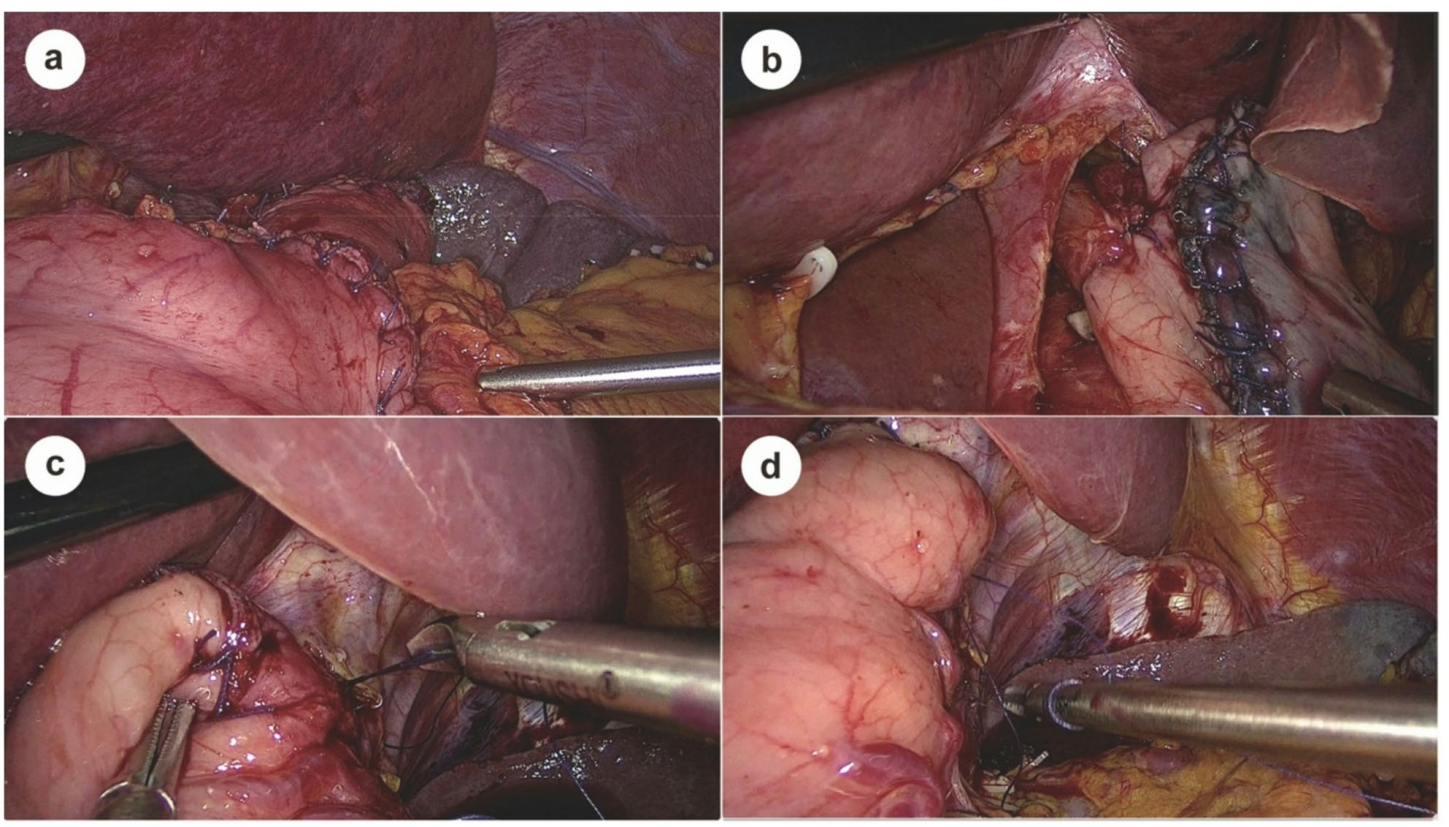
Laparoscopic confirmation of anatomical restoration following completion of two-suture biomimetic esophagogastric reconstruction. **a** Laparoscopic view of the anterior gastric wall after interrupted seromuscular suturing; imbrication at both ends shortens the anterior wall relative to the posterior wall, reconstructing the gastric angle. **b** Right-sided fixation between the remnant stomach and the esophageal wall establishes the high-pressure zone at the esophagogastric junction. **c** Left-sided fixation between the remnant stomach and the lateral esophageal wall reconstructs the angle of His and forms the neofundus, completing the circumferential configuration. **d** Final appearance of the neofundus after completion of bilateral fixation, forming a flap-valve-like structure and a functional high-pressure zone at the esophagogastric junction

In light of these considerations, we sought to explore a reconstruction approach that could incorporate key physiological anti-reflux features while maintaining minimal technical complexity. Based on this rationale, we introduced a simplified biomimetic technique using two-stitch fixation (Fig. 5b and c). Anatomically, this approach is intended to approximate the angle of His, the gastric angle, and a neofundus (Fig. 5d), thereby capturing several components of the physiological anti-reflux mechanism within a technically accessible framework. The central design concept lies in a two-suture strategy that gently modifies the local geometry to resemble a flap-valve configuration around the esophagogastric junction. These sutures may help restore a sharper angle at the junction and create a relatively higher-pressure zone surrounding the anastomosis during gastric filling.

As the remnant stomach expands, the suturing pattern also creates a small neofundus that provides compliant outward support against the cardia. This additional buttress may complement the flap-valve–like geometry by stabilizing the junction during episodes of increased intragastric pressure.

The gastric angle plays an important physiological role in directing gastric emptying. Its characteristic J-shaped contour separates the proximal reservoir from the antral pump, channels luminal flow toward the pylorus, and reduces pressure transmission toward the cardia [23, 24]. By serving as the boundary between the gastric body and antrum, it enables the antrum to function as an independent contractile unit that propels contents distally rather than back toward the esophagogastric junction. Inspired by these physiological features, we incorporated a simple maneuver to recreate an artificial gastric angle by inverting the seromuscular layers at both ends of the anterior gastric wall incision during closure. This shortens the anterior wall relative to the posterior wall and generates a contour that approximates the natural angle (Fig. 5a). In combination with the neofundus and the flap-valve–like geometry around the anastomosis, this artificial angle may act synergistically during gastric filling to reduce retrograde flow.

All of these adjustments—including the two anchoring sutures and the formation of an artificial gastric angle—were introduced with the intention of keeping the procedure as simple and practical as possible. They add only a modest, manageable step to the operation, do not require any specialized instruments, and do not place additional tension on the anastomosis. Although these refinements may be easier to incorporate than anti-reflux reconstructions that rely on multiple anastomoses, flap constructions, or pouch formations [22, 25], their broader value may lie in providing an option that remains technically accessible in settings with limited resources. The extent to which these modifications influence postoperative reflux outcomes will need to be clarified through studies with larger cohorts and appropriate comparative designs.

In this study, the modified reconstruction was associated with acceptable operative time, low intraoperative blood loss, and an uncomplicated postoperative course in most patients. Patients generally resumed ambulation within approximately 1–2 days and achieved gastrointestinal recovery shortly thereafter, with an average postoperative hospital stay of 7 days. Endoscopic evaluation during follow-up revealed no reflux esophagitis or anastomotic stricture. Taken together, these findings suggest that the technique is feasible and well tolerated, offering a practical option for postoperative anti-reflux management.

Although postoperative improvements in WHOQOL-BREF scores were observed—particularly in the social and environmental domains—these changes are best understood within the broader context of recovery after curative treatment. Relief of cancer-related symptoms and the gradual restoration of oral intake naturally contribute to better well-being, and a generally smooth postoperative course may further strengthen patients’ sense of security during rehabilitation. In the Chinese clinical context, many patients experience considerable preoperative anxiety and often regard successful tumor removal as a decisive turning point toward recovery. This psychological transition—from fear and uncertainty before surgery to a renewed sense of safety afterward—may substantially influence emotional and social functioning. In this light, while the QOL improvements reflect a culturally influenced psychological reassurance, a postoperative course free from reflux-related discomfort may have further supported this positive psychosocial transition by providing a stable physiological backdrop for recovery.

The volume of the remnant fundus may also influence postoperative reflux [25], as adequate preservation helps maintain gastric compliance and a more stable intragastric pressure environment. In our practice, precise preoperative endoscopic delineation of resection margins—using dye staining or titanium-clip marking for early lesions—allows safe tumor removal while retaining functional gastric volume (Supplem his preservation may complement the physiological intent of the modified reconstruction.

In summary, the modified esophagogastric reconstruction showed good safety, technical simplicity, and acceptable tolerance in this cohort. The approach preserves the convenience of conventional esophagogastrostomy while integrating anatomical refinements that may help reduce reflux. These characteristics indicate that the technique may serve as a practical option for gastrointestinal reconstruction following proximal gastrectomy. However, these findings should be regarded as preliminary. The small sample size, the absence of a comparative group, and the potential for patient-specific variability substantially limit the external validity of the results and the strength of any inferences that can be drawn. Although early postoperative findings are reassuring, reflux symptoms can still emerge or evolve over longer periods, and traditional assessment tools—such as endoscopy and barium studies—may not fully capture the functional subtleties required in contemporary practice [26]. Accordingly, larger, controlled studies with extended follow-up and more refined physiological assessment will be essential to determine the durability and true clinical value of this reconstruction.

## Conclusion

The modified esophagogastrostomy offers a technically simple and reproducible reconstruction strategy that incorporates key anatomical features relevant to reflux prevention. In this consecutive series, the approach was well tolerated, with smooth early postoperative recovery and reassuring early endoscopic and clinical findings. Because the technique requires no specialized instruments and adds only minimal operative complexity, it may be readily adopted across a range of surgical settings, including resource-limited centers. Further validation in larger, comparative cohorts with extended follow-up and objective physiologic assessment is needed to clarify its long-term performance.

## Supplementary Information

Supplementary Material 1.

Supplementary Material 2.

## Data Availability

All relevant data are included in this article. Additional de-identified data are available from the corresponding author on reasonable request and with approval from the institutional research center/ethics committee.

## References

[1] Bray F, Laversanne M, Sung H, Ferlay J, Siegel RL, Soerjomataram I, et al. Global cancer statistics 2022: GLOBOCAN estimates of incidence and mortality worldwide for 36 cancers in 185 countries. CA Cancer J Clin. 2024;74(3):229–63. 10.3322/caac.21834. 38572751 10.3322/caac.21834

[2] Urabe M, Matsusaka K, Ushiku T, Fukuyo M, Rahmutulla B, Yamashita H, et al. Adenocarcinoma of the stomach and esophagogastric junction with low DNA methylation show poor prognoses. Gastric Cancer. 2023;26(1):95–107. 10.1007/s10120-022-01344-3. 36224483 10.1007/s10120-022-01344-3

[3] Vial M, Grande L, Pera M. Epidemiology of adenocarcinoma of the esophagus, gastric cardia, and upper gastric third. Recent Results Cancer Res. 2010;182:1–17. 10.1007/978-3-540-70579-6_1. 20676867 10.1007/978-3-540-70579-6_1

[4] Chapelle N, Manfredi S, Lepage C, Faivre J, Bouvier AM, Jooste V. Erratum to: trends in gastric cancer incidence: a period and birth cohort analysis in a well-defined French population. Gastric Cancer. 2016;19(2):682. 10.1007/s10120-015-0548-2. 26445943 10.1007/s10120-015-0548-2

[5] Laparoscopic Surgery Group of the Endoscopist Branch in the Chinese Medical Doctor Association (CMDA); Chinese Esophagogastric Adenocarcinoma Research Collaboration Group (CEARC). Chinese society for diseases of the esophagus (CSDE); Chinese expert consensus on the surgical treatment for adenocarcinoma of esophagogastric junction (Edition 2024). Zhonghua Wei Chang Wai Ke Za Zhi. 2024;27(2):109–26. 38413076 10.3760/cma.j.cn441530-20231212-00213

[6] Japanese Gastric Cancer Association. Japanese gastric cancer treatment guidelines 2021 (6th edition). Gastric Cancer. 2023;26(1):1–25. 10.1007/s10120-022-01331-8. 36342574 10.1007/s10120-022-01331-8 PMC9813208

[7] Honda M, Yasunaga H, Michihata N, Miyakawa T, Kumazawa R, Matsui H, et al. Impact of guideline recommendation for novel surgical procedures on surgeons’ decisions: a time series analysis of gastric cancer surgeries from a nationwide cohort study. Int J Surg. 2023;109(3):316–22. 10.1097/JS9.0000000000000179. 36913310 10.1097/JS9.0000000000000179 PMC10389369

[8] He J, Chen WQ, Li ZS, Li N, Ren JS, Tian JH, et al. China guideline for the screening, early detection and early treatment of gastric cancer (2022, Beijing). Zhonghua Zhong Liu Za Zhi. 2022;44(7):634–66. 10.3760/cma.j.cn112152-20220617-00430. 35880331 10.3760/cma.j.cn112152-20220617-00430

[9] Yamashita H, Seto Y, Sano T, Makuuchi H, Ando N, Sasako M. Results of a nation-wide retrospective study of lymphadenectomy for esophagogastric junction carcinoma. Gastric Cancer. 2017;20(Suppl 1):69–83. 10.1007/s10120-016-0663-8. 27796514 10.1007/s10120-016-0663-8

[10] Khalayleh H, Kim YW, Man Yoon H, Ryu KW, Kook MC. Evaluation of lymph node metastasis among adults with gastric adenocarcinoma managed with total gastrectomy. JAMA Netw Open. 2021;4(2):e2035810. 10.1001/jamanetworkopen.2020.35810. 33566106 10.1001/jamanetworkopen.2020.35810 PMC7876588

[11] Kurokawa Y, Takeuchi H, Doki Y, Mine S, Terashima M, Yasuda T, et al. Mapping of lymph node metastasis from esophagogastric junction tumors: a prospective nationwide multicenter study. Ann Surg. 2021;274(1):120–7. 10.1097/SLA.0000000000003499. 31404008 10.1097/SLA.0000000000003499

[12] Davis JL, Ripley RT. Postgastrectomy syndromes and nutritional considerations following gastric surgery. Surg Clin North Am. 2017;97(2):277–93. 10.1016/j.suc.2016.11.005. 28325187 10.1016/j.suc.2016.11.005

[13] Michael S, Marom G, Brodie R, Salem SA, Fishman Y, Shein GS, et al. The angle of his as a measurable element of the anti-reflux mechanism. J Gastrointest Surg. 2023;27(11):2279–86. 10.1007/s11605-023-05808-4. 37620664 10.1007/s11605-023-05808-4

[14] Zhang R, Li Z, Li C, Ji F, Han X, Wang Z. Effect of laparoscopic angle of his reconstruction in the treatment of patients with gastroesophageal reflux disease and hiatal hernia. Chin Med J (Engl). 2022;135(14):1750–2. 10.1097/CM9.0000000000002211. 35947076 10.1097/CM9.0000000000002211 PMC9509176

[15] Yamashita Y, Yamamoto A, Tamamori Y, Yoshii M, Nishiguchi Y. Side overlap esophagogastrostomy to prevent reflux after proximal gastrectomy. Gastric Cancer. 2017;20(4):728–35. 10.1007/s10120-016-0674-5. 27942874 10.1007/s10120-016-0674-5

[16] Japanese Gastric Cancer Association. Japanese gastric cancer treatment guidelines 2014 (ver. 4). Gastric Cancer. 2017;20(1):1–19. 10.1007/s10120-016-0622-4. 10.1007/s10120-016-0622-4 PMC5215069 27342689

[17] Hölscher AH, Law S. Esophagogastric junction adenocarcinomas: individualization of resection with special considerations for Siewert type II, and Nishi types EG, E = G and GE cancers. Gastric Cancer. 2020;23(1):3–9. 10.1007/s10120-019-01022-x. 31691875 10.1007/s10120-019-01022-x

[18] Cao F, Hu C, Xu ZY, Zhang YQ, Huang L, Chen JH, et al. Current treatments and outlook in adenocarcinoma of the esophagogastric junction: a narrative review. Ann Transl Med. 2022;10(6):377. 10.21037/atm-22-1064. 35433931 10.21037/atm-22-1064 PMC9011222

[19] Wang ZY, Wang JT, Li RX, Wang GJ, Zhu TY, Gao BL. Effects of proximal gastrectomy with narrow gastric tube anastomosis compared with total gastrectomy with Roux-en-Y anastomosis on upper gastric cancer. Langenbecks Arch Surg. 2023;408(1):141. 10.1007/s00423-023-02878-5. 37020087 10.1007/s00423-023-02878-5

[20] Irino T, Ohashi M, Hayami M, Makuuchi R, Ri M, Sano T, et al. Updated review of proximal gastrectomy for gastric cancer or cancer of the gastroesophageal junction. J Gastric Cancer. 2025;25(1):228–46. 10.5230/jgc.2025.25.e12. 39822177 10.5230/jgc.2025.25.e12 PMC11739649

[21] Hosogi H, Yoshimura F, Yamaura T, Satoh S, Uyama I, Kanaya S. Esophagogastric tube reconstruction with stapled pseudo-fornix in laparoscopic proximal gastrectomy: a novel technique proposed for Siewert type II tumors. Langenbecks Arch Surg. 2014;399(4):517–23. 10.1007/s00423-014-1163-0. 24424495 10.1007/s00423-014-1163-0

[22] Ko HJ, Kim KH, Lee SH, Choi CW, Kim SJ, In Choi C, et al. Can proximal gastrectomy with double-tract reconstruction replace total gastrectomy? A propensity score matching analysis. J Gastrointest Surg. 2020;24(3):516–24. 10.1007/s11605-019-04195-z. 30937710 10.1007/s11605-019-04195-z

[23] Ferrua MJ, Singh RP. Modeling the fluid dynamics in a human stomach to gain insight of food digestion. J Food Sci. 2010;75(7):R151–62. 10.1111/j.1750-3841.2010.01748.x. 21535567 10.1111/j.1750-3841.2010.01748.x PMC2992692

[24] Pal A, Indireshkumar K, Schwizer W, Abrahamsson B, Fried M, Brasseur JG. Gastric flow and mixing studied using computer simulation. Proc Biol Sci. 2004;271(1557):2587–94. 10.1098/rspb.2004.2886. 15615685 10.1098/rspb.2004.2886 PMC1691895

[25] Li L, Cai X, Liu Z, Mou Y, Wang Y. Digestive tract reconstruction after laparoscopic proximal gastrectomy for gastric cancer: a systematic review. J Cancer. 2023;14(16):3139–50. 10.7150/jca.87315. 37859825 10.7150/jca.87315 PMC10583589

[26] Katz PO, Dunbar KB, Schnoll-Sussman FH, Greer KB, Yadlapati R, Spechler SJ. ACG clinical guideline for the diagnosis and management of gastroesophageal reflux disease. Am J Gastroenterol. 2022;117(1):27–56. 10.14309/ajg.0000000000001538. 34807007 10.14309/ajg.0000000000001538 PMC8754510

