# Supplementary material for: Dual-suture fundoplication for anti-reflux reconstruction after proximal gastrectomy: a single-center retrospective case series
Source: BMC Surg. 2026 Jan 26;26:150. doi: 10.1186/s12893-026-03499-7 (PMC12918137; doi:10.1186/s12893-026-03499-7)
Supplement: Supplementary file 1 — Supplementary Material 1. [file 12893_2026_3499_MOESM1_ESM.docx]

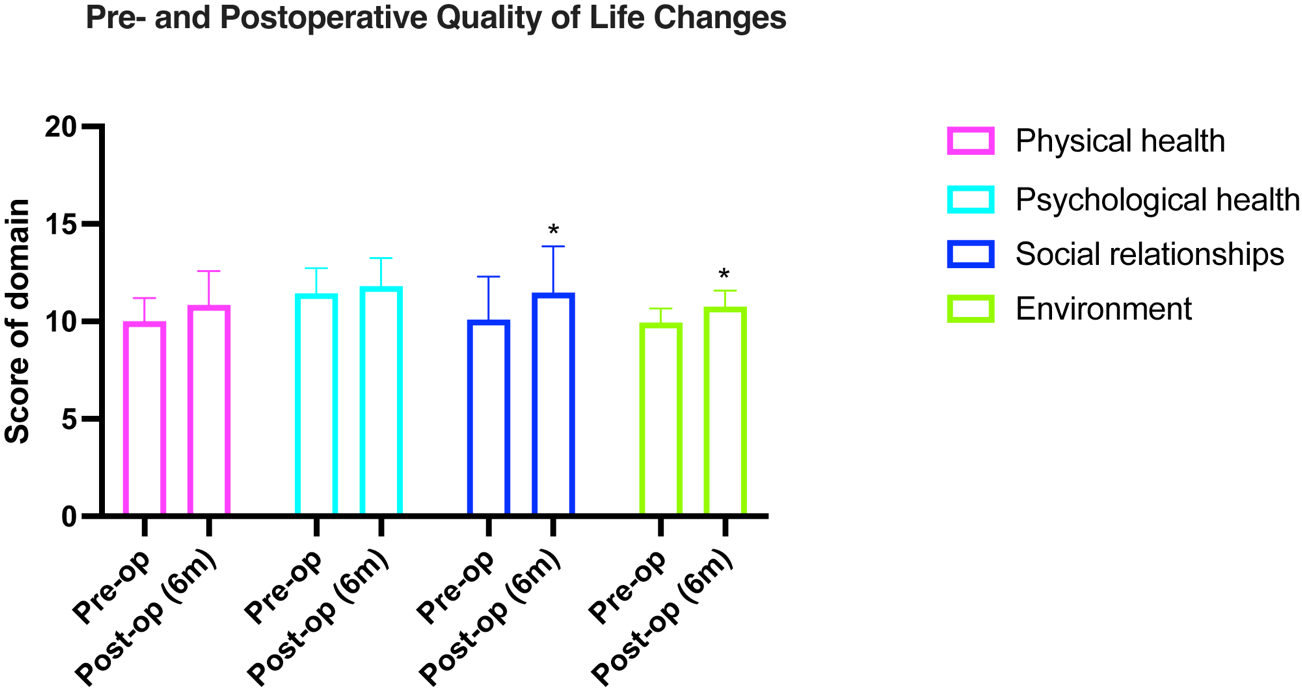


**Supplementary Figure 1.** Quality of life before and after surgery assessed by WHOQOL-BREF. Higher scores indicate better outcomes in each domain. Higher scores indicate better quality of life in the respective domain. *P* < 0.05 was considered statistically significant. * indicates significant differences between pre- and postoperative scores.


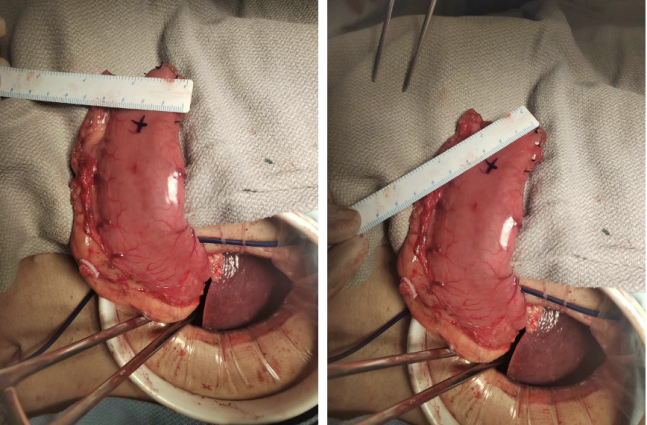


**Supplementary Figure 2.** The size of reconstructed artificial gastric fundus.
